# Erythrocyte Saturated Fatty Acids and Incident Type 2 Diabetes in Chinese Men and Women: A Prospective Cohort Study

**DOI:** 10.3390/nu10101393

**Published:** 2018-10-01

**Authors:** Jie-sheng Lin, Hong-li Dong, Geng-dong Chen, Zhan-yong Chen, Xiao-wei Dong, Ju-sheng Zheng, Yu-ming Chen

**Affiliations:** 1 Guangdong Provincial Key Laboratory of Food, Nutrition and Health; Department of Medical Statistics & Epidemiology, School of Public Health, Sun Yat-sen University, Guangzhou 510080, China; linjsh6@mail2.sysu.edu.cn (J.-s.L.); dhljiyi@163.com (H.-l.D.); chgengd@163.com (G.-d.C.); markname9@163.com (Z.-y.C.); dongxw@mail2.sysu.edu.cn (X.-w.D.); 2 Institute of Basic Medical Sciences, Westlake Institute for Advanced Study, Westlake University, Hangzhou 310024, China; zhengjusheng@wias.org.cn

**Keywords:** erythrocyte, saturated fatty acids, prospective cohort, type 2 diabetes

## Abstract

The association between circulating saturated fatty acids (SFAs) and incident type 2 diabetes (T2D) is reported in Western populations with inconsistent results, while evidence from Asian populations is scarce. We aimed to examine the associations between erythrocyte SFAs and incident T2D in a Chinese population. Between 2008 and 2013, a total of 2683 participants, aged 40–75 years, free of diabetes were included in the present analyses. Incident T2D cases were ascertained during follow-up visits. Gas chromatography was used to measure erythrocyte fatty acids at baseline. The Cox proportional hazards model was used to estimate the hazard ratios (HRs) and 95% confidence intervals (CIs). During 13,508 person years of follow-up, 216 T2D cases were identified. Compared with the first quartile, multivariable-adjusted HRs (95% CIs) of the fourth quartile were 1.20 (0.82–1.76; *p* = 0.242) for myristic acid (14-carbon tail, zero double bonds; 14:0), 0.69 (0.48–0.99; *p* = 0.080) for palmitic acid (16:0), 1.49 (1.02–2.19; *p* = 0.047) for stearic acid (18:0), 1.46 (1.00–2.12; *p* = 0.035) for arachidic acid (20:0), 1.48 (0.99–2.22; *p* = 0.061) for behenic acid (22:0), and 1.08 (0.74–1.56; *p* = 0.913) for lignoceric acid (24:0). Our findings indicate that individual erythrocyte SFAs are associated with T2D in different directions, with 18:0 and 20:0 SFAs positively associated with the risk, whereas no convincing inverse association for 16:0 SFAs.

## 1. Introduction

Diabetes was a global health problem in the past decade, with an estimated 451 million adults with the disease in 2017 globally [1]. Diet is one of the most important modifiable lifestyle factors for the prevention of type 2 diabetes (T2D), the predominant type of diabetes. The role of dietary saturated fatty acids (SFAs) in the prevention of T2D is still not clear and under debate [2,3]. Circulating SFAs reflect both dietary intake and endogenous synthesis [4], and investigation of their associations with T2D may provide new insight into the role of SFAs in diabetes etiology.

There is an ongoing interest in the associations of circulating SFAs with incident T2D, although inconclusive results are reported [5,6,7,8,9,10,11,12,13,14] (Table S1). Results from the European Prospective Investigation into Cancer and Nutrition (EPIC)-InterAct study suggested that plasma phospholipid myristic acid (14-carbon tail, zero double bonds; 14:0), palmitic acid (16:0), and stearic acid (18:0) were positively associated with incident T2D [5], which was consistent with several other prospective cohort studies [6,7,8]. In contrast, other prospective cohort studies found no association [10,11,12,13]. Similarly, results of studies that investigated the associations between very-long-chain SFAs (VLCSFAs, SFAs with 20 carbon atoms or more) and incident T2D were also inconsistent [5,8,9,12,14]. For example, two prospective studies reported that plasma phospholipid arachidic acid (20:0), behenic acid (22:0), and lignoceric acid (24:0) were inversely associated with incident T2D [5,14], while another study reported null association of erythrocyte 20:0, 22:0, and 24:0 SFAs with incident T2D [8].

Of note, all the above studies were exclusively conducted in Western populations, while there was one small nested case-control study among a Japanese population (*n* = 1014), which reported a null association of serum 14:0, 16:0, 18:0, or 20:0 SFAs with incident T2D [15].

The aim of the present study was to examine the associations of individual circulating SFAs with incident T2D in a community-based prospective cohort in southern China. As a secondary objective, we aimed to examine the associations of different groups of circulating SFAs with incident T2D, given the strong evidence from the EPIC-InterAct study about the diverse associations of different SFA groups [5].

## 2. Materials and Methods

### 2.1. Study Population

This study was based on the Guangzhou Nutrition and Health Study (GNHS), a community-based prospective cohort study designed to investigate the nutritional determinants of chronic diseases such as cardiovascular diseases, diabetes, and bone health. Between 2008 and 2013, participants were recruited via advertisements, health talks, and referrals in urban Guangzhou, China. Volunteers who lived in Guangzhou for more than five years, aged 40–75 years, were eligible for inclusion. GNHS included two batches of participant recruitment following the same criteria between 2008 and 2010 (*n* = 3169), and between 2012 and 2013 (*n* = 879), with a total of 4048 participants. All participants were followed up approximately every three years, and up to 31 May 2017, two follow-up visits were conducted for participants recruited between 2008 and 2010, and one follow-up visit for participants recruited between 2012 and 2013.

Participants were excluded (*n* = 1365) based on the following pre-defined criteria:

(1) a history of a self-reported cancer (*n* = 19) and chronic renal dysfunction (*n* = 4);
(2) diabetes at baseline (*n* = 323);
(3) without measurement of erythrocyte fatty acids (FAs) (*n* = 392);
(4) extreme dietary total energy intake (men, <800 kcal/day or >4000 kcal/day; women, <500 kcal/day or >3500 kcal/day; *n* = 35);
(5) diet variables (*n* = 41) or fasting glucose (*n* = 80) data missing;
(6) loss of follow-up (*n* = 471, 85% follow-up rate).

**Figure 1:**
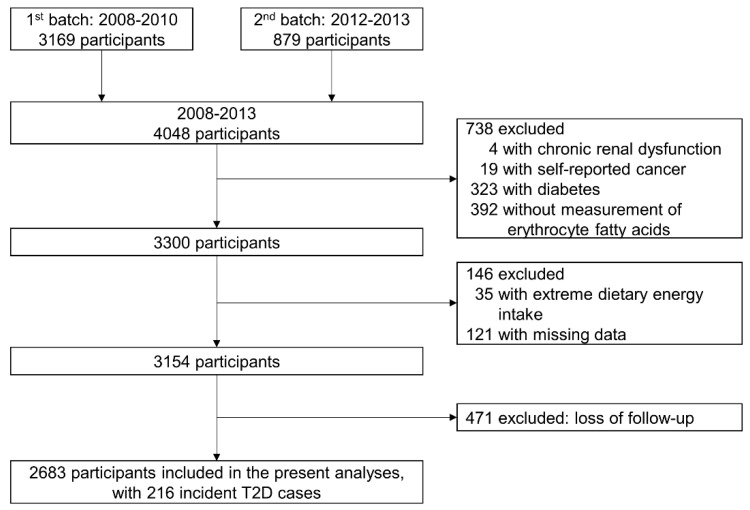
Flowchart of study participants.

Finally, 2683 participants were included in the present analyses, with a median 5.6 years of follow-up. The flowchart is presented in Figure 1. The study protocol of GNHS was registered (NCT03179657, ClinicalTrials.gov) and approved by the Ethics Committee of the School of Public Health at Sun Yat-sen University, and all participants provided written informed consent.

### 2.2. Data Collection

Face-to-face interviews were conducted in each survey by trained investigators. Information on socio-demographic characteristics (e.g., age, sex, household income, and education level), lifestyle (e.g., smoking, alcohol, and tea drinking), physical activity [16], and history of chronic diseases and medications was collected. A validated food frequency questionnaire was used to estimate the habitual dietary intakes over the past year [17]. Weight and height were measured with the participants wearing light clothing but shoes-off. Participants also provided venous blood after an overnight fasting. Serum, plasma, and erythrocytes were separated and stored at −80 °C until used for analysis.

### 2.3. Ascertainment of Incident T2D

Incident cases of T2D were identified as fasting glucose ≥7.0 mmol/L or glycated hemoglobin ≥6.5% (*n* = 157), or as self-reported diabetic medications (*n* = 59) during the follow-up visits according to the American Diabetes Association criteria for diagnosis and classification of diabetes [18], and incident T2D was ascertained up until 31 May 2017.

### 2.4. Measurement of FAs Relative Composition in Erythrocyte

Erythrocyte FAs at baseline were measured by gas chromatography. In brief, erythrocytes were hemolyzed and erythrocyte FA methyl esters were obtained as previously described [19]. FA methyl esters were separated by gas chromatographic analysis (7890 GC, Agilent, Guangzhou, China; DB-23 capillary column: 60 m × 0.25 mm, internal diameter × 0.15 mm film, Agilent). Individual FAs were identified by comparison with standard substances (Nu-Chek Prep, Inc., Waterville, MN, USA). Relative concentrations of individual FAs were calculated as a percentage of total FAs. Intra-assay coefficients of variation for 42 randomly selected duplicates were 17.3% for 14:0 SFAs, 3.3% for 16:0 SFAs, 6.3% for 18:0 SFAs, 6.4% for 20:0 SFAs, 14.4% for 22:0 SFAs, and 7.1% for 24:0 SFAs.

### 2.5. Measurement of Biochemical Parameters

Serum triglycerides (TG), high-density lipoprotein cholesterol (HDL-C), low-density lipoprotein cholesterol (LDL-C), and fasting glucose levels were determined with colorimetric methods using a Roche cobas 8000 c702 automated analyzer (Roche Diagnostics GmbH, Shanghai, China). The intra-assay coefficients of variation were 5.8% for TG, 4.3% for HDL-C, 3.1% for LDL-C, and 2.5% for fasting glucose levels. Glycated hemoglobin was measured by high-performance liquid chromatography using the Bole D-10 Hemoglobin A1c Program on a Bole D-10 Hemoglobin Testing System, and the intra-assay coefficient of variation was 0.75% for glycated hemoglobin.

### 2.6. Statistical Analysis

We examined the difference in baseline characteristics by future T2D cases and non-cases using the *t*-test or Mann–Whitney test for continuous variables, and the chi-square test for categorical variables. Spearman’s rank correlation coefficients were calculated between individual erythrocyte SFAs, and between food groups and erythrocyte SFAs.

The Cox proportional hazards model was used to estimate the hazard ratios (HRs) and 95% confidence intervals (CIs) of incident T2D across the second through fourth quartiles (compared with the first quartile) of erythrocyte SFAs under three statistical models to minimized the possibility of reverse causality. Model 1, adjusted for baseline age, sex, body mass index (BMI), and ratio of waist to hip circumference; Model 2, model 1 plus smoking status, alcohol drinking, tea drinking, education level, household income, physical activity, family history of diabetes, and total energy intake; Model 3, model 2 plus blood lipid and glycemic markers (TG, HDL-C, LDL-C, and fasting glucose).

As a secondary aim, we added three additional exposures based on groupings of even-chain SFAs (14:0 + 16:0 + 18:0) and VLCSFAs (20:0 + 22:0 + 24:0) and total SFAs, and examined their prospective associations with T2D using the above three models.

We also examined the interaction of erythrocyte SFAs with several pre-defined variables: age (continuous), sex, and BMI (continuous), by including interaction terms in the above final model (i.e., model 3), and we presented the stratified analyses if a significant interaction (*p* < 0.05) was found.

We also conducted several sensitivity analyses based on model 3. We accounted results for individual FAs independent of even-chain SFAs or VLCSFAs. For 14:0, 16:0, and 18:0 SFAs, we additionally adjusted for VLCSFAs; for 20:0, 22:0, and 24:0 SFAs, we additionally adjusted for even-chain SFAs. We examined the influence of additional dietary variables (intake of fruits and vegetables, sugar, dairy products, and red and processed meat) or erythrocyte unsaturated fatty acids (16:1 *n*-7, 18:1 *n*-9, 20:1 *n*-9, 22:1 *n*-9, 24:1 *n*-9; *n*-3 FAs: 18:3, 20:3, 20:5, 22:5, 22:6; *n*-6 FAs: 18:2, 18:3, 20:4) on the results. We also repeated analyses after excluding T2D cases (*n* = 13) occurring within one year after baseline to examine the potential influence of reverse causality.

All statistical analyses were conducted by using IBM SPSS software version 23.0 (SPSS, IBM, New York, NY, USA). A two-tailed *p*-value <0.05 was considered as statistically significant.

## 3. Results

Table 1 presents the baseline characteristic by future T2D cases and non-cases. At baseline, future cases aged 59.0 (5.7) years were older than non-cases aged 57.9 (5.7) years. Future cases had higher baseline BMI, ratio of waist to hip circumference, serum fasting glucose, and TG levels, and lower household income, dairy intake, and HDL-C levels, and were more likely to have a family history of diabetes compared with non-cases. Table 2 suggested that erythrocyte SFAs were significantly correlated with each other (*p* < 0.05), and 14:0, 16:0, and 18:0 SFAs were inversely correlated with 22:0 and 24:0 SFAs. The Spearman’s rank correlation coefficients between 16:0 and 22:0 SFAs was −0.298. The correlations of erythrocyte SFAs with dietary intakes are presented in Table S2.

During 13,508 person years of follow-up, 216 cases of incident T2D were identified. There were no associations among erythrocyte 14:0 and 24:0 SFAs and T2D incidence across the three models. After adjustment for baseline sociodemographic and lifestyle factors (model 1 and model 2), no association of 16:0 SFAs with T2D was observed. While after further adjustment for circulating lipid and glycemic markers in model 3, an inverse association for 16:0 SFAs was found with a HR of 0.69 (95% CIs: 0.48–0.99; *p* = 0.080) at the fourth quartile compared with the first quartile. In the multivariable-adjusted model (i.e., model 3), higher 18:0 and 20:0 SFA levels were associated with a higher risk of T2D (*p* < 0.05 for each) and higher 22:0 SFA levels tended to be associated with a higher risk of T2D (*p* = 0.061). Compared with the first quartile, the final (i.e., model 3) adjusted HRs (95% CIs) in the highest quartile (fourth quartile, Q4) were 1.49 (1.02–2.19) for 18:0 SFAs, 1.46 (1.00–2.12) for 20:0 SFAs, and 1.48 (0.99–2.22) for 22:0 SFAs (Table 3). There was no substantial difference in various sensitivity analyses (Table S4).

**Figure 2:**
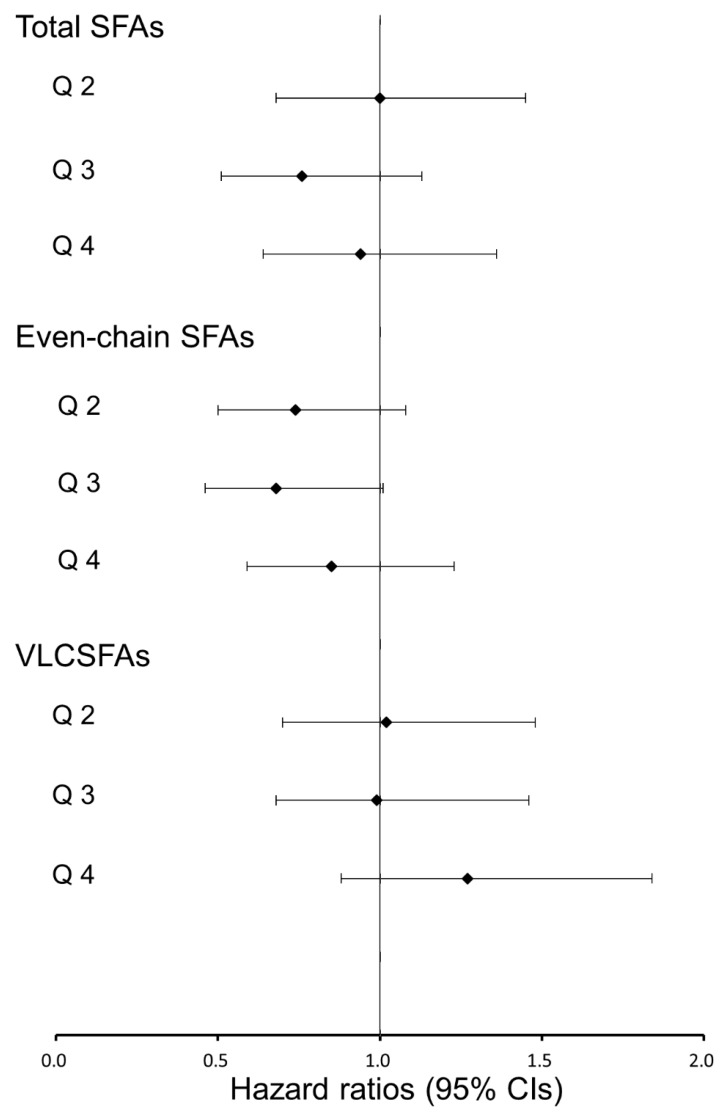
Hazard ratios (95% confidence intervals, CIs) for associations between erythrocyte saturated fatty acids (SFAs) and incident type 2 diabetes. The Cox proportional hazards model was used to estimate the hazard ratios and 95% CIs across the second through fourth quartiles (compared with the first quartile) after adjusted for potential confounders (i.e., model 3); even-chain SFAs, sum of 14:0, 16:0, and 18:0 SFAs; VLCSFAs, very-long-chain SFAs, sum of 20:0, 22:0, and 24:0 SFAs.

We did not find any significant association of groupings of erythrocyte total SFAs, even-chain SFAs and VLCSFAs with incident T2D (Figure 2, Table S3).

Circulating total SFAs significantly interacted with BMI (*p* = 0.025), and 14:0 SFAs interacted with sex (*p* = 0.037) for the risk of incident T2D. The results of stratified analysis by BMI for total SFAs or sex for 14:0 SFAs are presented in Figure S1. There was a positive association of 14:0 SFAs with incident T2D among men, but not women.

## 4. Discussion

In this prospective study of Chinese men and women, we investigated the associations of six individual and several groupings of erythrocyte SFAs with T2D incidence. We found that higher levels of 18:0 and 20:0 SFAs were associated with a higher risk of T2D, whereas there was no convincing association for 16:0 and 22:0 SFAs, and no association for 14:0 and 24:0 SFAs, or different groupings of SFAs.

Circulating 16:0 SFAs are the most abundant SFAs among total FA composition [5,20]. In the past decade, a positive association of circulating 16:0 SFAs with T2D was reported in several prospective cohort studies, including the EPIC-InterAct Study [5], the Atherosclerosis Risk in Communities Study [7], and the Cardiovascular Health Study [21]. Null association of circulating 16:0 SFAs with T2D was reported in several other prospective cohorts [10,12,15]. In contrast, results from 1346 Finnish men suggested a positive association of erythrocyte 16:0 SFAs with insulin sensitive index, but no association with T2D [11]. The inconsistent results across these above studies may be because of the diverse backgrounds of ethnicities and dietary habits in these study participants. Indeed, there are only two prospective studies among Asian populations reporting the 16:0–T2D association, including our present one and a Japanese cohort [15], and neither found a positive association. The discrepancy between the prior evidence and our present results may be because of the difference in dietary habits and dietary fat compositions. The majority of prior studies came from Western populations who consumed higher levels of high-fat products characterized by a high SFA content, and where the dominant SFAs in the diet are 16:0 SFAs [20,22]. In addition, the diverse intake of other dietary components, such as alcohol or carbohydrates, among Western and Asian populations may contribute to the inconsistent results, as these dietary components are also know to affect blood 16:0 SFA levels [23,24].

On the other hand, the inconsistent results may be attributed to FAs measured in different blood components. Positive associations of circulating 16:0 SFAs with T2D were more observed in plasma phospholipids than in erythrocytes (Table S1). Researchers, using the EPIC-Norfolk study, estimated the prospective association of different measures of FAs with incident T2D, and they observed greater magnitude and strength of association for FAs measured in plasma phospholipids than in erythrocytes [25], which may also partially explain our overall null results.

After 16:0 SFAs, 18:0 SFAs are the second-most abundant SFAs among total FA composition [5,20]. Similarly, results of studies that examined the associations between 18:0 SFAs and T2D were also inconsistent [5,7,8,9,21,26] (Table S1). Nevertheless, our present results are consistent with reports from several large cohort studies, including the Melbourne Collaborative Cohort Study case-cohort study [26], the EPIC-InterAct study [5], and the Atherosclerosis Risk in Communities Study [7].

So far, the prospective association between VLCSFAs and T2D is less well investigated, with a few studies showing an inverse association of circulating VLCSFAs with T2D in Western populations [5,12,14], while another study reported a positive association of erythrocyte 24:0 SFAs with T2D risk [9] (Table S1). Although we did not find a positive association of erythrocyte 24:0 SFAs with T2D, we did observe a positive association of erythrocyte 20:0 SFAs with T2D in our present study. VLCSFAs have cholesterol-raising potential, and plasma phospholipid VLCSFAs were positively associated with total cholesterol [27], which was an independent risk factor for T2D [28]. In vivo experiments suggested that VLCSFA accumulation in macrophages might enhance inflammatory and oxidative responses and play a role in the pathogenesis of inflammatory diseases [29].

VLCSFAs are primarily derived from limited foods, such as macadamia nuts and peanuts [20]. Findings from the Nurses’ Health Study suggested that higher nut and peanut consumption was inversely associated with T2D incidence [30], and it was reported that Western populations consumed 4–5 times the number of nuts and peanuts compared with that of Chinese populations [31], resulting in the inconsistent associations between our findings and other reports. More investigations are needed to explore the reason for the inconsistency and the related mechanisms.

Long-chain SFAs, especially VLCSFAs, are the major backbone subspecies of ceramide [32], which is considered as a contributor to diabetes. Firstly, ceramide inhibits insulin signaling by inhibiting insulin-stimulated phosphorylation of insulin receptor substrate-1 [33,34] and blocking activation of protein kinase B [34,35,36], which stimulates the development of insulin resistance [37,38]. Secondly, ceramide suppresses insulin synthesis by reducing insulin messenger RNA (mRNA) levels in pancreatic β-cells [39,40]. Finally, ceramide mediates the extrinsic apoptotic pathway, as well as the intrinsic mitochondrial and intrinsic endoplasmic reticulum apoptotic pathway in pancreatic β-cells, which results in a reduction of insulin synthesis [41,42]. Moreover, a previous study found that ceramides with 18:0 and 22:0 FAs had a more pronounced apoptotic effect in pancreatic β-cells [43].

We found an interaction of 14:0 SFAs with sex, and 14:0 SFAs were positively associated with T2D risk in male participants, but not in female participants. It was reported that free 14:0 SFAs would reduce the amount of albumin-bound testosterone, the main source of bioavailable testosterone [44]. A meta-analysis indicated that lower testosterone levels are associated with higher risk of T2D in men, but with lower risk in women [45]. It is possible that 14:0 SFAs might mediate bioavailable testosterone levels in relation to the risk of T2D, which might explain the opposite associations of 14:0 SFAs with T2D risk between women and men; however, more investigations are needed.

This study has several strengths. Firstly, we used an objective measurement of erythrocyte FAs. Secondly, our present study had a large sample size, and, to the best of our knowledge, provided the first evidence on this topic among Chinese populations. Thirdly, we included a variety of diet, lifestyle, and socio-demographic factors, as well as circulating biomarkers, in our statistical models, and we performed several sensitivity analyses, all of which supported our findings.

Several limitations should be considered. Firstly, we did not measure erythrocyte odd-chain SFAs, including pentadecanoic acid (15:0) and heptadecanoic acid (17:0), used as biomarkers of dairy intake [46]. Our results show that future cases had a lower dairy intake compared with non-cases (Table 1), and a previous study suggested that dairy intake was associated with a lower risk of T2D [2]. Although we adjusted for dairy intake in sensitive analyses and observed robust results, it may still be a limitation that we could not adjust for circulating 15:0 and 17:0 SFAs.

Secondly, erythrocyte FAs were measured at baseline only and erythrocyte FA compositions may change over time, which may cause potential exposure misclassification. Thirdly, we did not conduct an oral glucose tolerance test to identify diabetes, which might lead to undiagnosed diabetes cases. Finally, although we adjusted for multiple potential confounders, residual confounding due to unmeasured or imprecisely measured confounders could not be excluded.

## 5. Conclusions

In conclusion, our findings indicated that individual erythrocyte SFAs were associated with T2D in different directions, and we found that higher levels of 18:0 and 20:0 SFAs were associated with a higher risk of incident T2D, whereas no convincing inverse association was found for 16:0 SFAs. These results suggested that individual SFAs have differential associations with T2D among Chinese populations. However, these results should be interpreted with caution because we did not adjust for dairy biomarkers, and these results were based on a single measurement at baseline. More investigations are warranted to replicate our findings.

## Figures and Tables

**Table 1:** Baseline characteristics of study participants.

Baseline Characteristics	Future Cases (*n* = 216)	Non-Cases (*n* = 2467)	*p*-Value
	Mean (SD) or Percentage		
Age (year)	59.0 (5.7)	57.9 (5.7)	0.004
Sex (% female)	67.6	69.9	0.483
Weight (kg)	63.0 (9.9)	58.5 (9.5)	<0.001
Height (cm)	159 (7.8)	159 (7.5)	0.609
Body mass index, BMI (kg/m^2^)	24.8 (3.0)	23.0 (3.0)	<0.001
Waist circumference (cm)	87.2 (8.7)	82.3 (8.8)	<0.001
Hip circumference (cm)	95.0 (6.1)	92.6 (5.7)	<0.001
Ratio of waist to hip circumference	0.92 (0.06)	0.89 (0.07)	<0.001
Smoker (%)	18.5	14.9	0.152
Alcohol drinker (%)	5.10	6.70	0.353
Tea drinker (%)	56.9	50.7	0.079
**Education level (%)**			0.050
≤9 years	35.6	27.8	
9–12 years	41.7	47.0	
>12 years	22.7	25.2	
**Household income** **(Yuan/month/person), (%)**			0.037
≤500	2.10	1.90	
500–1500	31.0	25.8	
1500–3000	50.5	56.7	
>3000	14.4	15.5	
Family history of diabetes (%)	15.7	9.90	0.007
Physical activity (MET·h/day)	41.7 (15.5)	41.6 (14.9)	0.909
Total energy intake (kcal/day)	1756 (540)	1776 (496)	0.575
Vegetable intake (g/day)	399 (183)	382 (247)	0.342
Fruit intake (g/day)	140 (107)	149 (111)	0.200
Whole-grain intake (g/day)	10.6 (11.4)	12.3 (20.7)	0.219
Nuts and seeds intake (g/day)	7.46 (11.6)	7.06 (10.2)	0.094
Oil intake (g/day)	16.8 (9.7)	15.7 (10.4)	0.121
Sugar intake (g/day)	3.14 (6.1)	3.02 (3.1)	0.631
Dairy intake (g/day)	14.9 (12.8)	17.6 (15.1)	0.012
Red and processed meat intake (g/day)	85.3 (55.9)	83.5 (53.1)	0.633
Fish intake (g/day)	49.6 (37.6)	56.5 (78.5)	0.206
Serum fasting glucose (mmol/L)	5.30 (0.8)	4.60 (0.6)	<0.001
Serum TG (mmol/L)	1.98 (1.4)	1.50 (1.1)	<0.001
Serum HDL-C (mmol/L)	1.25 (0.3)	1.41 (0.3)	<0.001
Serum LDL-C (mmol/L)	3.63 (0.9)	3.58 (0.9)	0.459
**Erythrocyte SFAs, % of total fatty acids**			
14:0	0.32 (0.24)	0.29 (0.15)	0.006
16:0	27.7 (3.7)	27.7 (3.6)	0.948
18:0	17.6 (2.4)	17.3 (2.3)	0.122
20:0	0.48 (0.24)	0.48 (0.24)	0.723
22:0	1.44 (0.65)	1.50 (0.65)	0.235
24:0	4.96 (1.4)	5.00 (1.3)	0.659
Even-chain SFAs	45.6 (5.5)	45.3 (5.2)	0.070
VLCSFAs	6.90 (1.8)	7.00(1.8)	0.728
Total SFAs	52.5 (5.5)	52.2 (5.3)	0.569

Abbreviations: 14-carbon tail, zero double bonds (14:0), myristic acid; 16:0, palmitic acid; 18:0, stearic acid; 20:0, arachidic acid; 22:0, behenic acid; 24:0, lignoceric acid; SD, Standard deviation; BMI, body mass index; HDL-C, high-density lipoprotein cholesterol; LDL-C, low-density lipoprotein cholesterol; MET, metabolic equivalent of task; SFAs, saturated fatty acids; TG, triglycerides; VLCSFAs, very-long-chain SFAs.

**Table 2:** Spearman’s rank correlation of erythrocyte saturated fatty acids (*n* = 2683) ^a^.

	14:0	16:0	18:0	20:0	22:0	24:0
14:0	1					
16:0	0.253 ***	1				
18:0	0.286 ***	0.310 ***	1			
20:0	0.135 ***	0.037	0.209 ***	1		
22:0	−0.070 ***	−0.298 ***	−0.050 *	0.374 ***	1	
24:0	−0.173 ***	−0.212 ***	−0.088 ***	0.328 ***	0.583 ***	1

Abbreviations: 14-carbon tail, zero double bonds (14:0), myristic acid; 16:0, palmitic acid; 18:0, stearic acid; 20:0, arachidic acid; 22:0, behenic acid; 24:0, lignoceric acid. ^a^ Spearman’s rank correlation coefficients were calculated between individual erythrocyte saturated fatty acids; * *p* < 0.05, *** *p* < 0.001.

**Table 3:** Association of erythrocyte saturated fatty acids (SFAs) with incident type 2 diabetes ^a^.

SFAs	Statistical Model	Quartiles (Q) of Erythrocyte SFA Concentrations				*p*-Value
		Q1 (*n* = 670)	Q2 (*n* = 671)	Q3 (*n* = 672)	Q4 (*n* = 670)	
14:0	Median (%)	0.19	0.24	0.29	0.40	
	Cases/person years	44/3298	47/3247	53/3408	72/ 555	
	Model 1 ^b^	1 (reference)	1.01 (0.67–1.52)	1.07 (0.72–1.60)	1.36 (0.93–1.98)	0.088
	Model 2 ^c^	1 (reference)	1.01 (0.67–1.53)	1.05 (0.70–1.57)	1.36 (0.93–1.98)	0.072
	Model 3 ^d^	1 (reference)	0.90 (0.59–1.35)	0.89 (0.59–1.33)	1.20 (0.82–1.76)	0.242
16:0	Median (%)	23.7	26.5	28.4	31.5	
	Cases/person years	62/3573	46/3256	47/3143	61/3535	
	Model 1 ^b^	1 (reference)	0.67 (0.45–0.98)	0.68 (0.46–1.00)	0.85 (0.59–1.21)	0.454
	Model 2 ^c^	1 (reference)	0.68 (0.46–1.00)	0.68 (0.46–1.00)	0.85 (0.60–1.22)	0.424
	Model 3 ^d^	1 (reference)	0.55 (0.37–0.81)	0.53 (0.35–0.78)	0.69 (0.48–0.99)	0.080
18:0	Median (%)	15.5	16.4	17.4	19.5	
	Cases/person years	45/3436	52/3304	54/3273	65/3495	
	Model 1 ^b^	1 (reference)	1.19 (0.79–1.77)	1.23 (0.82–1.82)	1.42 (0.97–2.08)	0.075
	Model 2 ^c^	1 (reference)	1.14 (0.77–1.71)	1.20 (0.81–1.79)	1.40 (0.96–2.05)	0.072
	Model 3 ^d^	1 (reference)	1.19 (0.80–1.79)	1.35 (0.91–2.02)	1.49 (1.02–2.19)	0.047
20:0	Median (%)	0.36	0.41	0.46	0.59	
	Cases/person years	58/3257	45/3333	57/3371	56/3546	
	Model 1 ^b^	1 (reference)	0.79 (0.54–1.17)	0.99 (0.68–1.42)	1.01 (0.70–1.46)	0.715
	Model 2 ^c^	1 (reference)	0.77 (0.53–1.15)	1.00 (0.69–1.44)	1.01 (0.70–1.46)	0.687
	Model 3 ^d^	1 (reference)	1.04 (0.70–1.54)	1.39 (0.96–2.02)	1.46 (1.00–2.12)	0.035
22:0	Median (%)	0.46	1.48	1.74	2.07	
	Cases/person years	57/3207	59/3363	55/3386	45/3552	
	Model 1 ^b^	1 (reference)	1.06 (0.74–1.53)	1.08 (0.75–1.57)	0.93 (0.63–1.39)	0.803
	Model 2 ^c^	1 (reference)	1.06 (0.73–1.53)	1.09 (0.75–1.58)	0.97 (0.65–1.45)	0.939
	Model 3 ^d^	1 (reference)	1.53 (1.05–2.22)	1.61 (1.10–2.36)	1.48 (0.99–2.22)	0.061
24:0	Median (%)	3.86	4.53	5.09	6.18	
	Cases/person years	57/3392	53/3242	52/3328	54/3546	
	Model 1 ^b^	1 (reference)	0.97 (0.67–1.41)	0.94 (0.64–1.36)	1.04 (0.71–1.51)	0.923
	Model 2 ^c^	1 (reference)	1.01 (0.70–1.47)	0.99 (0.68–1.44)	1.08 (0.74–1.58)	0.755
	Model 3 ^d^	1 (reference)	0.99 (0.68–1.44)	0.99 (0.68–1.44)	1.08 (0.74–1.56)	0.913

Abbreviations: 14-carbon tail, zero double bonds (14:0), myristic acid; 16:0, palmitic acid; 18:0, stearic acid; 20:0, arachidic acid; 22:0, behenic acid; 24:0, lignoceric acid; BMI, body mass index; CI, confidence interval; HDL-C, high-density lipoprotein cholesterol; HR, hazard ratio; LDL-C, low-density lipoprotein cholesterol; SFAs, saturated fatty acids; TG, triglycerides. ^a^ Multivariable-adjusted hazard ratios (95% CIs) were calculated for Q2 to Q4 of the erythrocyte SFAs compared with Q1. ^b^ Model 1: adjusted for age, sex, BMI, and ratio of waist to hip circumference. ^c^ Model 2: included covariates in model 1 plus smoking status, alcohol drinking, tea drinking, education level, household income, physical activity, family history of diabetes, and total energy intake. ^d^ Model 3: included covariates in model 2 plus LDL-C, HDL-C, TG, and fasting glucose levels.

## Supplementary Materials

The following are available online at http://www.mdpi.com/2072-6643/10/10/1393/s1. Table S1: Review of literature examining association of circulating SFAs with incident type 2 diabetes. Table S2: Spearman’s rank correlation of erythrocyte saturated fatty acids and dietary intakes (*n* = 2683). Table S3: Association of groupings of erythrocyte SFAs with incident type 2 diabetes. Table S4: Sensitivity analyses for association of erythrocyte SFAs with incident type 2 diabetes. Figure S1: Associations of erythrocyte SFAs with incident type 2 diabetes, stratified by categories of BMI and sex.
