# Supplementary material for: Erythrocyte Saturated Fatty Acids and Incident Type 2 Diabetes in Chinese Men and Women: A Prospective Cohort Study
Source: Nutrients. 2018 Oct 1;10(10):1393. doi: 10.3390/nu10101393 (PMC6212875; doi:10.3390/nu10101393)
Supplement: Supplementary file 1 [file nutrients-10-01393-s001.pdf]

Supplementary Materials

Erythrocyte Saturated Fatty Acids and Incident Type 2 Diabetes in Chinese Men and Women: A Prospective Cohort Study

Table S1. Review of literature examining association of circulating SFAs with incident type 2 diabetes.

Author, Year	Age range	N[a]	Years of follow-up	Biological samples	14:0	16:0	18:0	20:0	22:0	24:0	Total SFAs
Wang, 2003[1]	45–64 y	2909 (252)	9.0 y	Plasma PL	—[b]	↑	↑	—	—	—	↑
Hodge, 2007[2]	36–72 y	346/3391	4.0 y	Plasma PL	—	NS	↑	—	—	—	↑
Krachler, 2008[3]	40–60 y	159/291	5.4 y	Erythrocyte	NS	NS	NS	—	—	—	NS
Patel, 2010[4]	40–79 y	199/184	10.0 y	Erythrocyte	NS	↑	NS	—	—	—	NS
Kroger, 2011[5]	35–65 y	673/2114	7.0 y	Erythrocyte	NS	NS	NS	↓	NS	↑	NS
Alhazmi, 2014[6]	55–85 y	37/150	5.0 y	Whole blood	—	NS	NS	—	—	↓	—
Forouhi, 2014[7]	53.7 y	12132/15919	9.8 y	Plasma PL	↑	↑	↑	↓	↓	↓	—
Mahendran, 2014[8]	45–73 y (men)	735 (30)	5.0 y	Erythrocyte	—	NS	NS	—	—	—	NS
Lankinen, 2015[9]	45–68 y (men)	1302 (71)	5.9 y	Plasma PL	NS	NS	↑	NS	NS	NS	NS
Lemaitre, 2015[10]	≥65 y	3179 (284)	10.0 y	Plasma PL	—	—	—	↓	↓	↓	—
Ma, 2015[11]	≥65 y	3004 (297)	10.0 y	Plasma PL	NS	↑	↑	—	—	—	—
Harris, 2016[12]	65–80 y (women)	6379 (703)	11.0 y	Erythrocyte	↑	↑	NS	NS	NS	NS	NS
Takkunen, 2016[13]	40–65 y	383 (155)	11.0 y	Serum	NS	NS	NS	—	—	—	NS
Yakoob, 2016[14]	30–75 y	3333 (277)	15.2 y	Plasma PL	NS	—	—	—	—	—	—
Akter, 2017[15]	34–69 y	336/678	5.0 y	Serum	NS	NS	NS	NS	—	—	NS

Abbreviations: 14:0, myristic acid; 16:0, palmitic acid; 18:0, stearic acid; 20:0, arachidic acid; 22:0, behenic acid; 24:0, lignoceric acid; SFAs, saturated fatty acids; PL, phospholipids. [a] Cohort sample size (incident cases) or cases/controls. [b] "—", no data; " ↑ ", positive association; " ↓ ", inverse association; "NS", nonsignificant.

Table S2. Spearman's rank correlation of erythrocyte SFAs and dietary intake (N = 2683) [a.]

	14:0	16:0	18:0	20:0	22:0	24:0
Vegetable	0.065**	−0.021	−0.004	0.059**	0.062**	−0.005
Fruit	0.013	−0.028	−0.022	0.004	0.041*	0.023
Whole grain	0.019	−0.014	−0.008	0.040*	0.030	0.024
Nuts and seeds	−0.037	−0.010	−0.034	0.032	0.060**	0.053**
Animal and vegetable oil	0.045*	−0.030	−0.006	0.042*	0.020	0.045*
Alcohol	−0.045*	−0.004	−0.032	−0.061**	−0.053**	−0.017
Dairy products	0.066**	−0.014	−0.001	0.048*	0.027	0.015
Sugar	0.025	−0.043*	−0.007	0.046*	0.067**	0.033
Red and processed meat	0.045*	−0.025	0.019	0.007	0.036	0.003
Fish	0.012	−0.019	−0.018	0.010	0.036	0.002

Abbreviations: 14:0, myristic acid; 16:0, palmitic acid; 18:0, stearic acid; 20:0, arachidic acid; 22:0, behenic acid; 24:0, lignoceric acid. [a] Spearman's rank correlation coefficients were calculated between erythrocyte saturated fatty acids and dietary intake. * $p < 0.05$, ** $p < 0.01$, *** $p < 0.001$.

Table S3. Association of groupings of erythrocyte SFAs with incident type 2 diabetes [a.]

		Quartiles of erythrocyte SFAs concentrations				
		Q1 (n = 670)	Q2 (n = 671)	Q3 (n = 672)	Q4 (n = 670)	*p*-Value
Even-chain SFAs	Median (%)	40.2	43.4	45.6	50.8	
	Cases/ person years	58/ 3557	51/ 3235	46/ 3183	61/ 3532	
	Model 1[b]	1 (reference)	0.78 (0.53–1.14)	0.75 (0.51–1.11)	0.92 (0.64–1.32)	0.700
	Model 2[c]	1 (reference)	0.78 (0.53–1.14)	0.76 (0.51–1.12)	0.92 (0.64–1.32)	0.710
	Model 3[d]	1 (reference)	0.74 (0.50–1.08)	0.68 (0.46–1.01)	0.85 (0.59–1.23)	0.351
VLCSFAs	Median (%)	5.23	6.29	7.21	8.71	
	Cases/ person years	61/ 3265	53/ 3330	47/ 3383	55/ 3530	
	Model 1[b]	1 (reference)	0.84 (0.58–1.21)	0.81 (0.55–1.19)	0.98 (0.68–1.42)	0.866
	Model 2[c]	1 (reference)	0.85 (0.59–1.22)	0.84 (0.57–1.23)	1.01 (0.70–1.47)	0.997
	Model 3[d]	1 (reference)	1.02 (0.70–1.48)	0.99 (0.68–1.46)	1.27 (0.88–1.84)	0.399
Total SFAs	Median (%)	47.6	50.1	52.1	58.3	
	Cases/ person years	54/ 3533	58/ 3212	47/ 3181	57/ 3583	
	Model 1[b]	1 (reference)	1.00 (0.69–1.46)	0.81 (0.55–1.20)	0.96 (0.66–1.40)	0.609
	Model 2[c]	1 (reference)	0.99 (0.68–1.44)	0.82 (0.55–1.21)	0.96 (0.66–1.39)	0.619
	Model 3[d]	1 (reference)	1.00 (0.68–1.45)	0.76 (0.51–1.13)	0.94 (0.64–1.36)	0.395

Abbreviations: 14:0, myristic acid; 16:0, palmitic acid; 18:0, stearic acid; 20:0, arachidic acid; 22:0, behenic acid; 24:0, lignoceric acid; BMI, body mass index; CI, confidence interval; even-chain SFAs, sum of 14:0, 16:0, and 18:0; HDL-C, high-density lipoprotein cholesterol; HR, hazard ratio; LDL-C, low-density lipoprotein cholesterol; SFAs, saturated fatty acids; TG, triglycerides; VLCSFAs, very-long-chain SFAs, sum of 20:0, 22:0, and 24:0 SFAs. [a] Multivariable-adjusted hazard ratios (95% CIs) were calculated for second to fourth quartiles (Q2 to Q4) of the erythrocyte SFAs, compared with the first quartile (Q1). [b] Model 1: adjusted for age, sex, BMI, and ratio of waist to hip circumference; [c] Model 2: included covariates in model 1 plus smoking status, alcohol drinking, tea drinking, education level, household income, physical activity, family history of diabetes, and total energy intake; [d] Model 3: included covariates in model 2 plus LDL-C, HDL-C, TG, and fasting glucose levels.

Table S4. Sensitivity analyses for association of erythrocyte SFAs with incident type 2 diabetes [a].

		Quartiles of erythrocyte SFAs concentrations				
		Q1	Q2	Q3	Q4	*p*-Value
14:0	Model 3[b]	1 (reference)	0.90 (0.59–1.35)	0.89 (0.59–1.33)	1.20 (0.82–1.76)	0.242
	Model 3a[c]	1 (reference)	0.89 (0.59–1.35)	0.89 (0.59–1.33)	1.20 (0.82–1.76)	0.282
	Model 3b[d]	1 (reference)	0.90 (0.60–1.36)	0.89 (0.59–1.33)	1.22 (0.84–1.79)	0.214
	Model 3c[e]	1 (reference)	0.90 (0.59–1.36)	1.00 (0.66–1.52)	1.29 (0.56–2.80)	0.197
	Model 3d[f]	1 (reference)	0.88 (0.58–1.34)	0.91 (0.60–1.37)	1.17 (0.79–1.73)	0.344
16:0	Model 3[b]	1 (reference)	0.55 (0.37–0.81)	0.53 (0.35–0.78)	0.69 (0.48–0.99)	0.080
	Model 3a[c]	1 (reference)	0.55 (0.37–0.81)	0.53 (0.35–0.78)	0.69 (0.48–0.99)	0.114
	Model 3b[d]	1 (reference)	0.54 (0.37–0.80)	0.52 (0.35–0.77)	0.69 (0.48–0.99)	0.074
	Model 3c[e]	1 (reference)	0.60 (0.39–0.91)	0.60 (0.37–0.94)	0.84 (0.46–1.55)	0.289
	Model 3d[f]	1 (reference)	0.55 (0.36–0.82)	0.51 (0.34–0.77)	0.69 (0.47–0.99)	0.081
18:0	Model 3[b]	1 (reference)	1.19 (0.80–1.79)	1.35 (0.91–2.02)	1.49 (1.02–2.19)	0.047
	Model 3a[c]	1 (reference)	1.20 (0.80–1.79)	1.35 (0.91–2.02)	1.49 (1.02–2.19)	0.041
	Model 3b[d]	1 (reference)	1.19 (0.79–1.77)	1.34 (0.90–2.00)	1.50 (1.02–2.20)	0.046
	Model 3c[e]	1 (reference)	1.25 (0.83–1.89)	1.47 (0.96–2.24)	1.71 (1.02–2.86)	0.031
	Model 3d[f]	1 (reference)	1.22 (0.81–1.85)	1.33 (0.88–2.01)	1.55 (1.05–2.29)	0.040
20:0	Model 3[b]	1 (reference)	1.04 (0.70–1.54)	1.39 (0.96–2.02)	1.46 (1.00–2.12)	0.035
	Model 3a[c]	1 (reference)	1.04 (0.70–1.54)	1.39 (0.96–2.02)	1.46 (1.00–2.12)	0.028
	Model 3b[d]	1 (reference)	1.06 (0.71–1.57)	1.43 (0.99–2.09)	1.47 (1.01–2.14)	0.038
	Model 3c[e]	1 (reference)	1.04 (0.69–1.55)	1.39 (0.94–2.05)	1.60 (1.04–2.46)	0.018
	Model 3d[f]	1 (reference)	1.04 (0.69–1.56)	1.45 (0.99–2.12)	1.43 (0.97–2.11)	0.046
22:0	Model 3[b]	1 (reference)	1.53 (1.05–2.22)	1.61 (1.10–2.36)	1.48 (0.99–2.22)	0.061
	Model 3a[c]	1 (reference)	1.53 (1.05–2.22)	1.61 (1.10–2.36)	1.48 (0.99–2.22)	0.060
	Model 3b[d]	1 (reference)	1.52 (1.05–2.21)	1.61 (1.10–2.36)	1.50 (1.00–2.26)	0.055
	Model 3c[e]	1 (reference)	1.16 (0.68–1.99)	1.22 (0.69–2.15)	1.30 (0.71–2.38)	0.375
	Model 3d[f]	1 (reference)	1.54 (1.05–2.27)	1.67 (1.13–2.48)	1.45 (0.95–2.20)	0.095
24:0	Model 3[b]	1 (reference)	0.99 (0.68–1.44)	0.99 (0.68–1.44)	1.08 (0.74–1.56)	0.913
	Model 3a[c]	1 (reference)	0.99 (0.68–1.44)	0.99 (0.68–1.44)	1.08 (0.74–1.56)	0.942
	Model 3b[d]	1 (reference)	0.97 (0.67–1.42)	0.99 (0.68–1.44)	1.08 (0.74–1.57)	0.825
	Model 3c[e]	1 (reference)	1.05 (0.70–1.57)	1.04 (0.68–1.35)	1.22 (0.78–1.89)	0.397
	Model 3d[f]	1 (reference)	1.11 (0.75–1.63)	1.04 (0.70–1.54)	1.11 (0.75–1.65)	0.852
Even-chain SFAs	Model 3[b]	1 (reference)	0.72 (0.49–1.06)	0.68 (0.46–1.01)	0.85 (0.59–1.23)	0.351
	Model 3b[d]	1 (reference)	0.74 (0.50–1.08)	0.66 (0.45–0.98)	0.85 (0.59–1.22)	0.346
	Model 3c[e]	1 (reference)	0.80 (0.53–1.22)	0.79 (0.50–1.26)	0.98 (0.52–1.86)	0.720
	Model 3d[f]	1 (reference)	0.73 (0.50–1.09)	0.64 (0.42–0.97)	0.87 (0.60–1.26)	0.386
VLCSFAs	Model 3[b]	1 (reference)	1.02 (0.70–1.48)	0.99 (0.68–1.46)	1.27 (0.88–1.84)	0.399
	Model 3b[d]	1 (reference)	1.01 (0.70–1.47)	1.00 (0.68–1.46)	1.29 (0.89–1.86)	0.343
	Model 3c[e]	1 (reference)	0.88 (0.58–1.33)	0.73 (0.46–1.16)	1.08 (0.68–1.72)	0.773
	Model 3d[f]	1 (reference)	1.05 (0.72–1.55)	1.03 (0.69–1.53)	1.27 (0.87–1.87)	0.417
Total SFAs	Model 3[b]	1 (reference)	1.00 (0.68–1.45)	0.76 (0.51–1.13)	0.94 (0.64–1.36)	0.395
	Model 3b[d]	1 (reference)	0.98 (0.67–1.43)	0.76 (0.51–1.13)	0.85 (0.70–1.03)	0.397
	Model 3c[e]	1 (reference)	1.07 (0.71–1.60)	0.80 (0.50–1.27)	0.95 (0.48–1.87)	0.927
	Model 3d[f]	1 (reference)	0.97 (0.66–1.43)	0.78 (0.52–1.17)	0.94 (0.64–1.38)	0.459

Abbreviations: 14:0, myristic acid; 16:0, palmitic acid; 18:0, stearic acid; 20:0, arachidic acid; 22:0, behenic acid; 24:0, lignoceric acid; BMI, body mass index; CI, confidence interval; even-chain SFAs, sum of 14:0, 16:0, and 18:0; HDL-C, high-density lipoprotein cholesterol; HR, hazard ratio; LDL-C, low-density lipoprotein cholesterol; SFAs, saturated fatty acids; TG, triglycerides; VLCSFAs, very-long-chain SFAs, sum of 20:0, 22:0, and 24:0 SFAs. [a] Multivariable-adjusted hazard ratios (95% CIs) were calculated for Q2 to Q4 of the erythrocyte SFAs, compared with Q1. [b] Model 3: adjusted for age, sex, BMI, ratio of waist to hip circumference, smoking status, alcohol drinking, tea drinking, education level, household income, physical activity, family history of diabetes, total energy intake, LDL-C, HDL-C, TG, and fasting glucose levels; [c] Model 3a: for 14:0, 16:0, and 18:0 SFAs, adjusted for model 3 + VLCSFAs; for 20:0, 22:0 and 24:0 SFAs, adjusted for model 3 + even-chain SFAs; [d] Model 3b: adjusted for model 3 + fruits and vegetables, sugar, dairy products, and red and processed meat;. [e] Model 3c: adjusted for model 3 + erythrocyte monounsaturated fatty acids (16:1 n-7, 18:1 n-9, 20:1 n-9, 22:1 n-9, 24:1 n-9), erythrocyte polyunsaturated fatty acids (n-3 FA: 18:3, 20:3, 20:5, 22:5, 22:6; n-6 FA: 18:2, 18:3, 20:4); [f] Model 3d: repeated analyses of model 3 after excluding type 2 diabetes cases (n = 13) occurring within one year after baseline.

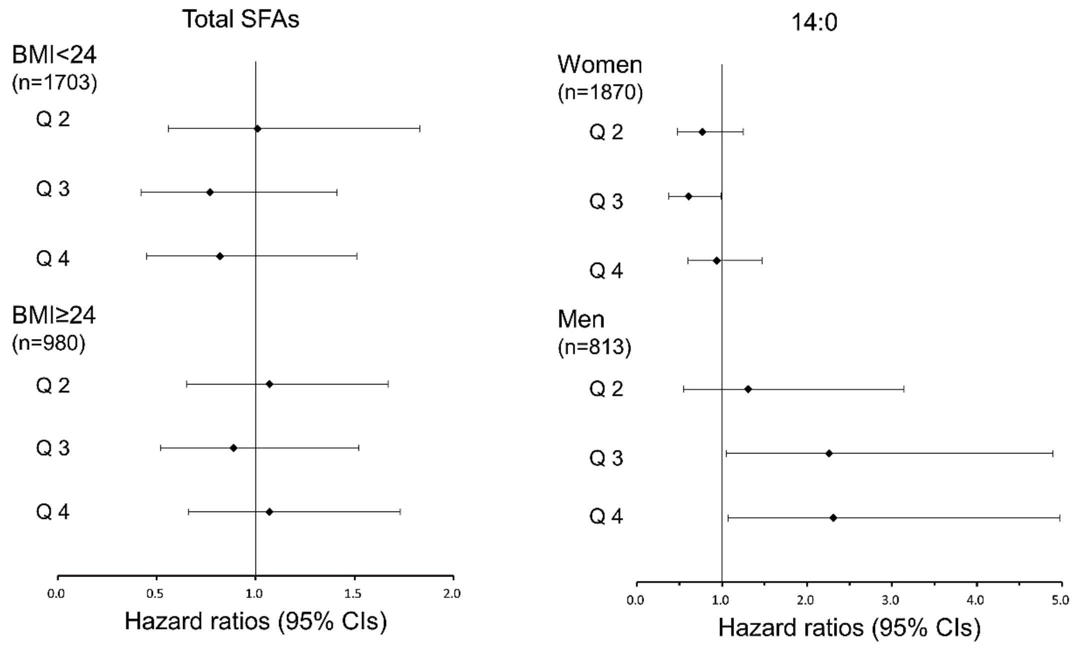

Figure S1. Associations of erythrocyte SFAs with incident type 2 diabetes, stratified by categories of body mass index (BMI) and sex. The Cox proportional hazards model was used to estimate the hazard ratios and 95% confidence intervals (CIs) across the second to fourth quartiles (compared with the first quartile) after being adjusted for age, sex, BMI, ratio of waist to hip circumference, smoking status, alcohol drinking, tea drinking, education level, household income, physical activity, family history of diabetes, and total energy intake, as well as low-density lipoprotein cholesterol, high-density lipoprotein cholesterol, triglyceride, and fasting glucose levels. We found interactions of total SFA levels with BMI ($p = 0.025$), and 14:0 SFAs with sex ($p = 0.037$).
